# Supplementary figures and images for: Transcriptome analysis of Chelidonium majus elaiosomes and seeds provide insights into fatty acid biosynthesis
Source: PeerJ. 2019 May 3;7:e6871. doi: 10.7717/peerj.6871 (PMC6501766; doi:10.7717/peerj.6871)

## A

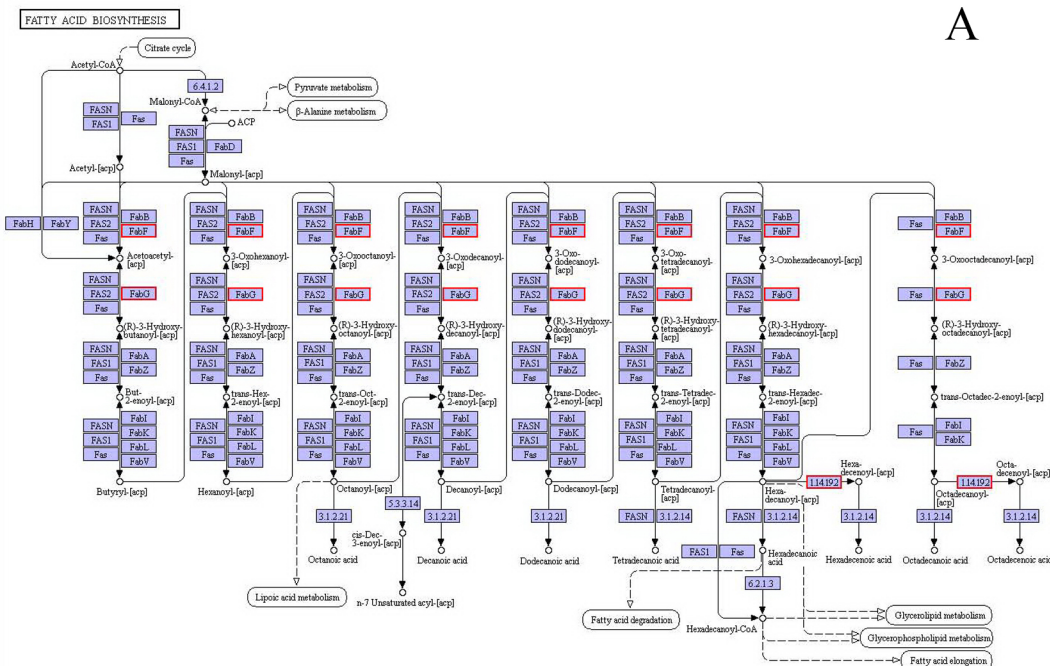

00061 8/13/15  
(c) Karehisa Laboratories

## B

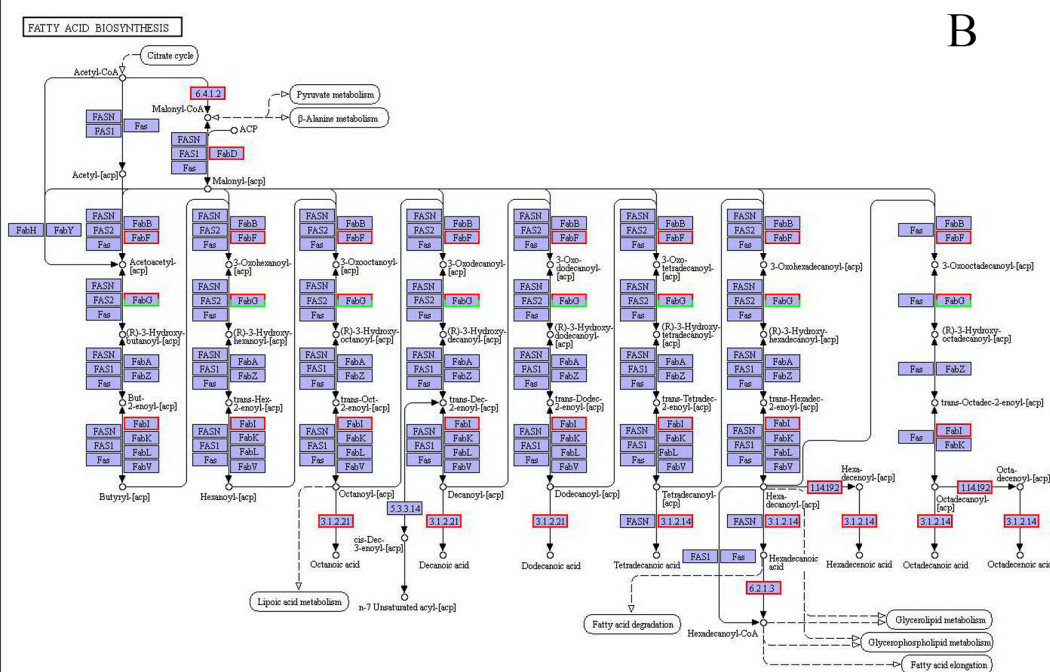

00061 8/13/15  
(c) Kanehisa Laboratories

## C

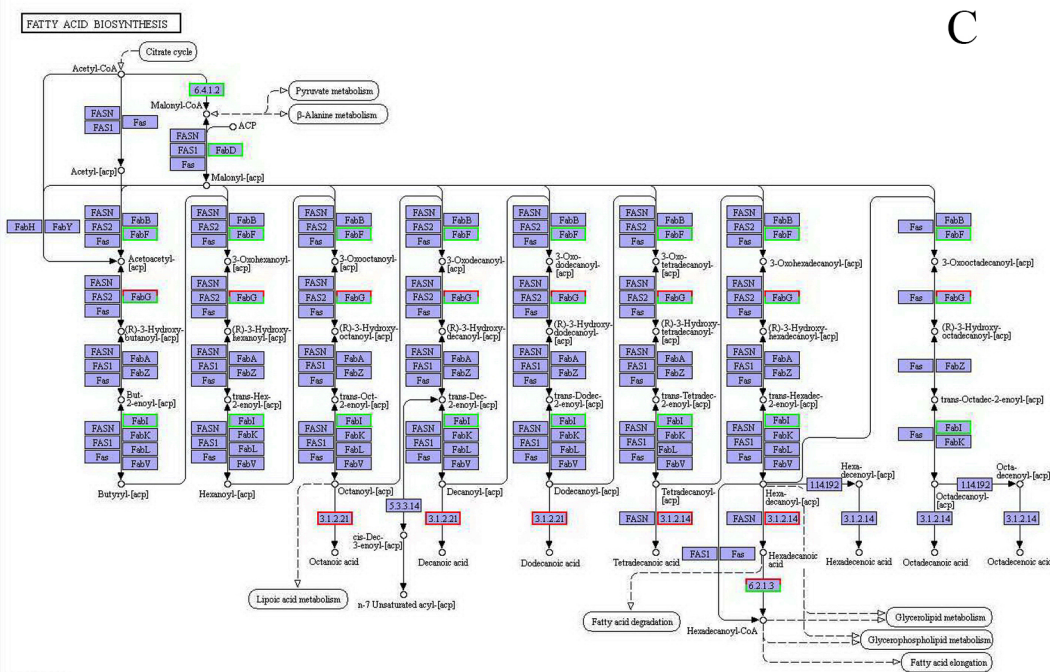

00061 8/13/15  
(c) Kanehisa Laboratories

Supplement: Supplemental Information 3 — Red represents up-regulated gene, green represents down-regulated gene. Overview of de novo fatty acid biosynthesis pathways: (A) Different genes expression between Ch01 and Ch02; (B) Different genes expression between Ch02 and Ch03; (C) Different genes expression between Ch03 and Ch04. Indentified enzymes include: ACCase, FatB, FabA, FatA/B, DesA1/2, ACSL. [file peerj-07-6871-s003.pdf]
